# Intraoperative Chemotherapy with a Novel Regimen Improved the Therapeutic Outcomes of Colorectal Cancer

**DOI:** 10.7150/jca.35450

**Published:** 2019-10-15

**Authors:** Zhihua Liu, Yifeng Zou, Yuming Rong, Xingyuan Shi, Chen Li, Chao Li, Yinghai Tian, Hongcheng Lin, Min Liu, Jinsheng Weng, Ting Liu, Xiaomei Li, Chao Lei, Weipeng Li, Xinke Zhou

**Affiliations:** 1 Department of Center Laboratory, the Fifth Affiliated Hospital of Guangzhou Medical University, Guangzhou, Guangdong, 510799, China;; 2 Department of Anorectal Surgery, the Fifth Affiliated Hospital of Guangzhou Medical University, Guangzhou, Guangdong, 510799, China;; 3 Department of Surgery, the Sixth Affiliated Hospital of Shanghai JiaoTong University, Shanghai, 200233, China;; 4 Department of Colorectal Surgery, the Sixth Affiliated Hospital, Sun Yat-sen University, Guangzhou, Guangdong, 510655, China;; 5 Department of VIP, Sun Yat-Sen University Cancer Center, Guangzhou, Guangdong, 510060, China.

**Keywords:** colorectal cancer, intraoperative chemotherapy, prognosis, therapy

## Abstract

**Background:** This study sought to evaluate the efficacy of a novel intraoperative chemotherapy (IOC) regimen that consists of hydroxycamptothecin, tumor necrosis factor (TNF), 5-fluorouracil (5-FU), and calcium folinate (CF) on the outcomes of colorectal cancer (CRC).

**Methods:** In total, 551 CRC patients who had undergone surgical resection were evaluated. Among these patients, 247 were treated with postoperative adjuvant chemotherapy, and 193 were treated with intraoperative chemotherapy. Of the CRC patients who underwent chemotherapy, 52 were treated with both postoperative adjuvant chemotherapy and intraoperative chemotherapy. Patients' characteristics, including age, sex, stage, differentiation, lymph node metastasis, surgical-pathological staging, tumor location, tumor size, and relapse-free survival, were collected.

**Results:** IOC for CRC therapy was associated with a more favorable survival prognosis (HR, 0.30, 95%CI, 0.19-0.48, P<0.001) independent of other clinical covariates. CRC patients treated with IOC survived longer than patients who were not treated with IOC did during surgery (P<0.0001, Kaplan-Meier log rank). Meanwhile, a Kaplan-Meier analysis demonstrated that individuals who received both IOC and POC survived longer than patients who received only POC: for stage II and stage III patients (P=0.0001, Kaplan-Meier log rank), stage II patients alone (P=0.02, Kaplan-Meier log rank), and stage III patients alone (P=0.046, Kaplan-Meier log rank).

**Conclusions:** The therapeutic effects of colorectal cancer by intraoperative chemotherapy with a novel regimen were enhanced, which improved the prognosis of patients with CRC.

## Introduction

Colorectal cancer (CRC) is the third most common cancer and the second most common cause of cancer-related mortality in Western countries, where there are approximately 600,000 new cases annually^1^. Furthermore, recent decades have witnessed a rapid increase in CRC morbidity in rapidly developing countries, such as China^2^. Currently, surgery is the mainstay of curative treatment; however, cancer recurrence and metastasis following surgery are common^3^. Therefore, there is an urgent need to further improve CRC treatment.

In an attempt to prevent cancer recurrence and metastasis following surgery, postoperative adjuvant intra-arterial chemotherapeutic agents have been used^4^-^7^. However, postoperative adjuvant intra-arterial chemotherapy does not significantly prolonged survival in all cases^8^-^9^. Intra-arterial chemotherapy is also associated with considerable complications and high costs^10^-^11^. Rashidi et al.^12^ have suggested a new strategy of intraoperative chemotherapy (IOC) for treating resectable highly malignant human colon cancer liver metastasis using *in vivo* experiments. The results of these studies have demonstrated that IOC is an effective and convenient treatment strategy to prolong the survival of rats.

In the present study, we evaluated the efficacy of IOC on the therapeutic outcomes of CRC using a novel regimen.

## Methods

### Patient selection

Between 2001 and 2008, 551 CRC patients who had undergone surgical resection were assessed for eligibility. Cases of familial adenomatous polyposis or human nonpolyposis CRC were excluded from this study. Patients' characteristics, including age, sex, disease stage, differentiation, lymph node metastasis, surgical-pathological staging, tumor location, tumor size, and relapse-free survival were collected. The final follow-up date for all of the cases was September 1, 2016.

### Criteria for inclusion

The following inclusion criteria were used for patients in this study: 1) age between 20 and 95 y; 2) a diagnosis confirmed by biopsy and histologic testing.

### Criteria for exclusion

The exclusion criteria were as follows: 1) pregnancy, 2) clinically significant immunodeficiency, 3) evidence of infection.

### Intraoperative chemotherapy

Intraoperative chemotherapy regimens consisting of hydroxycamptothecin, tumor necrosis factor (TNF), 5-fluorouracil (5-FU), and calcium folinate (CF) were administered during the operations. Hydroxycamptothecin was used for peritoneal irrigation during the operations, while TNF, 5-FU, and CF were given as intravenous injections.

### Postoperative adjuvant chemotherapy

The analyses of the response to postoperative adjuvant chemotherapy included only TNM stage II and III patients because TNM stage I patients have excellent survival prognoses regardless of therapy, and treatment in stage IV is palliative. The chemotherapy regimens were primarily fluorouracil-based, with or without leucovorin, levamisole, or cisplatin.

### Statistical Analysis

The statistical analyses were performed with GraphPad Prism software (GraphPad Prism Software, Version 5.01, GraphPad, San Diego, CA) and SPSS for Windows version 15.0.0 (SPSS, Inc., USA). Survival curves were generated using the Kaplan-Meier method, and the statistical analyses were performed using the log-rank test. Multivariate analyses were evaluated with Cox proportional hazards models. Statistical significance was defined as P < 0.05.

## Results

### Assembly of Tumor Samples

All of the 551 patients (100%) included in the study were Chinese. Patients with familial adenomatous polyposis or human nonpolyposis CRC were excluded from this study. In total, 247 (44.83%) patients were treated with postoperative adjuvant chemotherapy, and 193 (35.03%) patients were treated with intraoperative chemotherapy. Of the CRC patients who received chemotherapy, 52 were treated with both postoperative adjuvant chemotherapy and intraoperative chemotherapy. In the study, 303 (54.99%) patients were male and 248 (45.01%) were female. Detailed clinical characteristics are listed in **Table 1**.

### The association of IOC and POC with Prognosis for CRC patients

To determine whether the prognostic value of IOC was independent of other risk factors associated with clinical outcomes of CRC, a multivariate analysis was performed using the Cox proportional hazard model. The risk variables examined included IOC, POC, age, differentiation, lymph node metastasis, surgical-pathological staging, tumor location, and tumor size. These factors were generally known to significantly affect the outcome of CRC. In the univariate analysis, IOC (HR 0.33, 95%CI, 0.23-0.48, P<0.001), POC (HR 0.62, 95%CI, 0.45-0.87, P=0.006), TNM staging (HR 2.52, 95%CI, 1.89-3.36, P<0.001), lymph node metastasis (HR 1.70, 95%CI, 1.28-2.25, P<0.001), and differentiation (HR 1.92, 95%CI, 1.09-3.36, P=0.024) were significantly associated with survival, while age, gender, tumor location, and tumor size were not (**Table 2**). In the final multivariate Cox regression model, which included IOC, POC, TNM staging, lymph node metastasis, distant metastasis, tumor size, age, gender, differentiation, and tumor location, IOC for CRC therapy was associated with an improved survival prognosis (HR 0.30, 95%CI, 0.19-0.48, P<0.001) independent of other clinical covariates (**Table 2**).

### The association of IOC and POC with therapeutic outcomes for CRC patients

**Figure 1:**
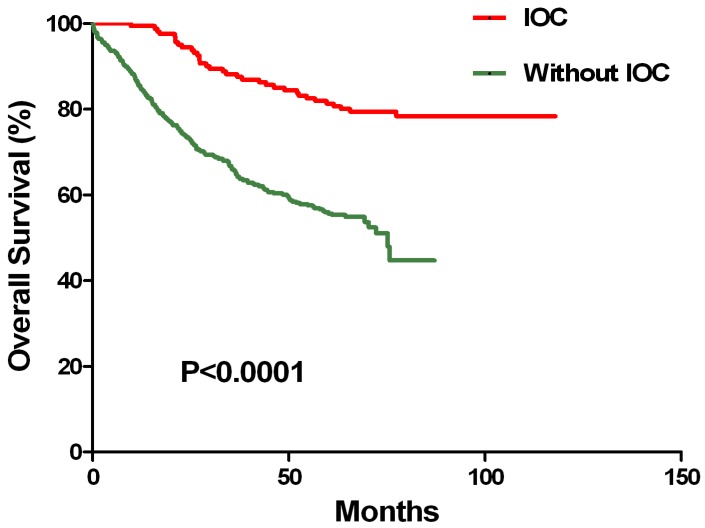
** Association of IOC with chemotherapy outcomes for CRC patients.** In the CRC patients, IOC was associated with longer survival, in the group that received chemotherapy, than was without IOC (P < 0.0001, Kaplan-Meier log rank).

To further analyze the CRC patient response to IOC, Kaplan-Meier analyses were performed. IOC regimens, which consisted of hydroxycamptothecin, TNF, 5-FU, and CF were administered during the operations. Hydroxycamptothecin was used as peritoneal irrigation during the operations, while TNF, 5-FU, and CF were given as intravenous injections. The results demonstrated that patients treated with IOC survived longer than the patients who were not treated with IOC during the surgery (P<0.0001, Kaplan-Meier log rank) (**Figure 1**).

**Figure 2:**
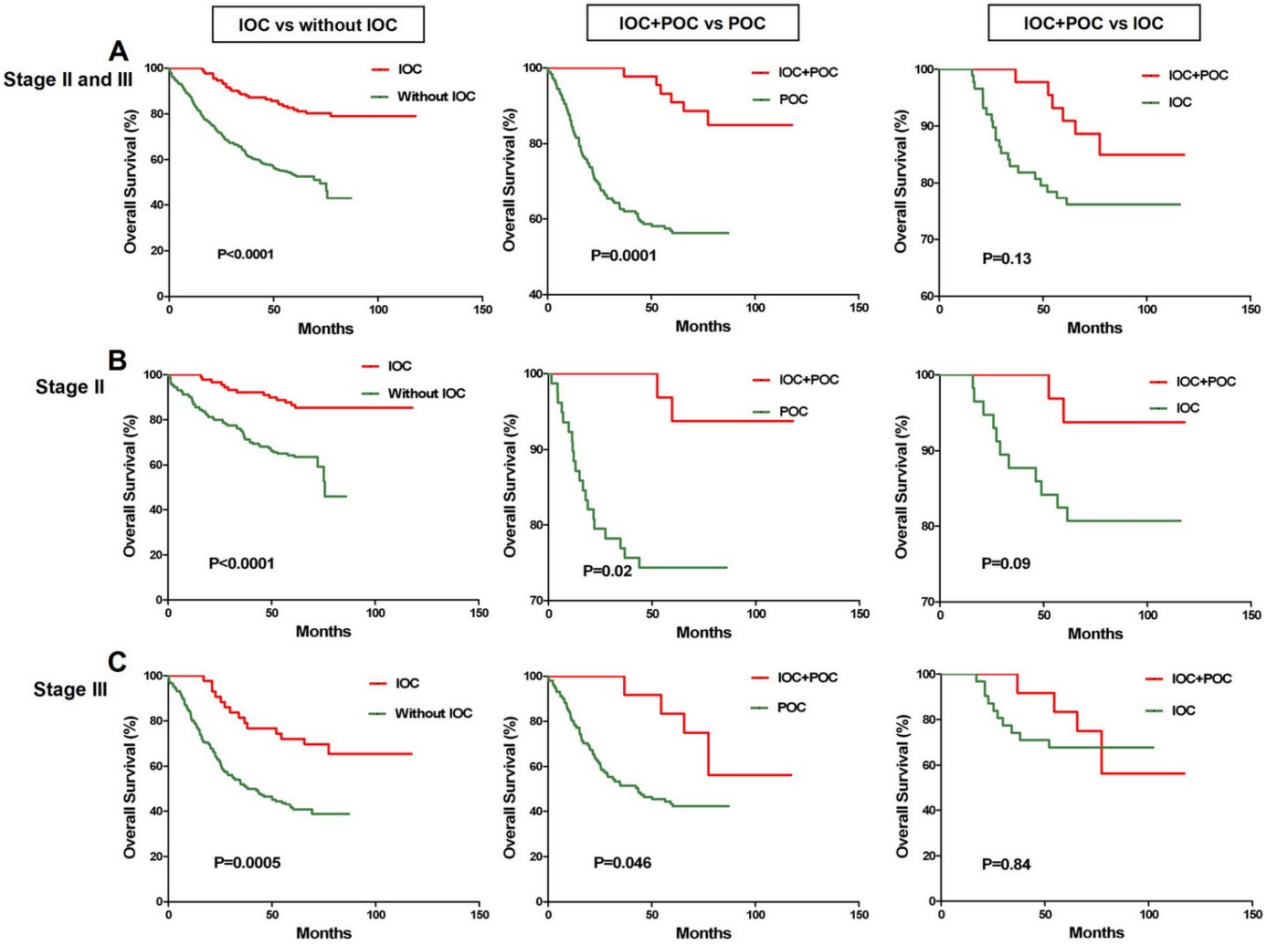
** Association of IOC and POC with chemotherapy outcomes for the CRC patients with TNM stage II or III. A.** For the 459 CRC stage II or III patients, IOC (n=153) was associated with a favorable prognosis (P<0.0001, Kaplan-Meier log rank); CRC patients who received both IOC and POC (n=52) survived longer than did patients who received only POC (n=195) (P=0.0001, Kaplan-Meier log rank). **B.** For the 267 CRC stage II patients, IOC (n=107) was associated with a favorable prognosis (P<0.0001, Kaplan-Meier log rank); CRC patients who received both IOC and POC (n=32) survived longer than the patients who only received POC (n=110) ( (P=0.02, Kaplan-Meier log rank). **C.** For the 192 CRC stage III patients, IOC (n=44) was associated with a favorable prognosis (P=0.0005, Kaplan-Meier log rank); CRC patients who received both IOC and POC (n=20) survived longer than the patients who received only POC (n=85) (P=0.046, Kaplan-Meier log rank).

Because the patients who received IOC might also receive POC after surgery, POC could affect the survival of these patients. This result could be caused by the IOC, POC, or a combination of both. An analysis of the response to postoperative adjuvant chemotherapy included only the TNM stage II and III patients because TNM stage I patients have excellent survival prognoses regardless of therapy, and treatment in stage IV is palliative. We analyzed the association of therapeutic outcomes in stage II and stage III CRC patients treated with postoperative adjuvant chemotherapy in the current study. The POC regimens were primarily fluorouracil based with or without leucovorin, levamisole, or cisplatin. The Kaplan-Meier analyses demonstrated that IOC was also associated with a favorable prognosis in stage II and stage III patients (P<0.0001, Kaplan-Meier log rank), stage II patients alone (P<0.0001, Kaplan-Meier log rank), and stage III patients alone (P=0.0005, Kaplan-Meier log rank) (**Figure 2**). The patients who received both IOC and POC survived longer than the patients who only received POC for stage II and stage III patients (P=0.0001, Kaplan-Meier log rank), stage II patients alone (P=0.02, Kaplan-Meier log rank), and stage III patients alone (P=0.046, Kaplan-Meier log rank), but not the patients who only received IOC for stage II and stage III patients (P=0.13, Kaplan-Meier log rank), stage II patients alone (P=0.09, Kaplan-Meier log rank), and stage III patients alone (P=0.84, Kaplan-Meier log rank) (**Figure 2**). Therefore, combining IOC and POC could significantly prolong the survival outcomes for CRC patients.

### Side effects

The side effects of IOC involves in perforation, bleeding, and infection, while no side effects occurred in our study. Of the 193 patients who suffered from the IOC, no IOC related complications were found.

## Discussion

Advances in diagnosis and surgical therapy have improved the prognosis of early CRC; however, the incidence of recurrence for Dukes' B and C CRC is approximately 50%^13^. The incidence of locoregional recurrence can been reduced significantly by total mesorectal excision^14^. The use of a similar approach, extensive lymph node resection, has been questioned on colon cancer^15^; surgery alone, even when macroscopically complete, is not adequate. In fact, microscopic residual disease is the cause of local recurrence in almost all patients^3^. To date, numerous adjuvant treatments have been used to prevent disease recurrence and improve survival^16^-^17^. Systemic chemotherapy has been shown to improve survival in stage III colon cancer^18^. Locoregional recurrence in locally advanced CRC is the result either of a tumor involving the serosa and perforation of the bowel wall or of the iatrogenic dissemination of cancer emboli that triggers locoregional tumors within 2-3 years^17^. If chemotherapy is administered with peritoneal irrigation and intravenous injection during an operation, microscopic residual tumors resulting from surgical manipulations in locally advanced colorectal cancer surgery may be eradicated.

In the present study, the clinical outcome was encouraging, and we observed no postoperative deaths. The postoperative morbidity rate was acceptable and comparable to that reported by other investigators 3, 19-20. The surgery-related complication rate can be considered similar to that observed in major surgery studies, which supports the hypothesis that IOC does not increase the risk of postoperative complications. The mild locoregional toxicity, together with the low rate of systemic adverse effects, strengthens our opinion that IOC is a safe procedure.

Zhou et al reported a study of short-term effect analysis of intraoperative intraperitoneal perfusion chemotherapy with lobaplatin for colorectal cancer, indicating no effect on short-term recovery in patients with CRC 21. However, survival times was not evaluated. Safety of intraoperative chemotherapy with 5-FU for colorectal cancer patients was also performed 22, and showed that it can be safely performed during colorectal surgery. In our study, we used novel regimen of hydroxycamptothecin, tumor necrosis factor (TNF), 5-fluorouracil (5-FU), and calcium folinate (CF) for IOC, and follow up study indicated a prognostic benefit.

Currently, it is known that IOC regimens consisting of hydroxycamptothecin, TNF, 5-FU, and CF for the treatment of CRC patients have a prognostic benefit. The current results demonstrated that CRC patients treated with IOC had longer survival times than the patients who were not treated with IOC during the surgery. In addition, we also analyzed the association of therapeutic outcomes in stage II and stage III CRC patients treated with postoperative adjuvant chemotherapy regimens that were primarily fluorouracil based with or without leucovorin, levamisole, or cisplatin. Kaplan-Meier analyses demonstrated that IOC was also associated with a favorable prognosis in stage II and stage III patients, stage II patients alone, and stage III patients alone. The patients who received both IOC and POC survived longer than the patients who only received POC for stage II and stage III (P=0.0001, Kaplan-Meier log rank), stage II patients alone, and stage III patients alone, but not the patients who only received IOC in stage II and stage III, stage II patients alone, and stage III patients alone. Therefore, a combination of both IOC and POC could significantly prolong survival for CRC patients. We conclude that the IOC was effective in CRC and that IOC+POC for stage II and stage III CRC patients was a predictor of long-term disease-free survival.

## Conclusions

In conclusion, the therapeutic effects of colorectal cancer by intraoperative chemotherapy with a novel regimen were enhanced, which improved the prognosis of the treated CRC patients. There is an urgent need for us to find a safer and more effective way to improve prognosis in further study of the IOC.

## Figures and Tables

**Table 1:** Clinical characteristics of colorectal cancer patients.

Clinical features	Number (%)^a^
**Gender - no. (%)**	
Female	248 (45.01)
Male	303 (54.99)
**Age - years**	
Median	68
Range	24-91
**Location - no. (%)^b^**	
Ascending colon	151 (27.40)
Transverse colon	24 (4.36)
Descending colon	48 (8.71)
Sigmoid colon	141 (25.59)
Rectum	182 (33.03)
Missing data	10 (1.81)
**TNM stage - no. (%)^c^**	
I	53 (9.62)
II	267 (48.46)
III	192 (34.85)
IV	22 (3.99)
Missing	17 (3.09)
**Pathological type - no. (%)**	
Tubular adenocarcinoma	442 (80.22)
Mucinous adenocarcinoma	57 (10.34)
Mixed adenocarcinoma	52 (9.44)
**Tumor size - no. (%)**	
< 5 cm	245 (44.46)
≥ 5 cm	250 (45.37)
Missing data	56 (10.16)
**Lymph node metastasis - no. (%)**	
Yes	201 (36.48)
No	337 (61.16)
Missing data	13 (2.36)
**Differentiation -no. (%)**	
Well	59 (10.71)
Moderate	356 (64.61)
Poor	136 (24.68)
**Postoperative adjuvant chemotherapy - no. (%)^d^**	
Yes	247 (44.83)
No	281 (51.00)
Missing data	23 (4.17)
**Intraoperative chemotherapy - no. (%)^e^**	
Yes	193 (35.03)
No	331 (60.07)
Missing data	27 (4.90)
**Survival - no. (%)**	
Median	63.97
Range	0.13 - 117.93

^a^Percentages may not sum to 100 because of rounding. ^b^Tumor location was available for 542 patients in the current study. Four patients had both rectal and sigmoid colon cancer, and 1 patient had both descending colon and sigmoid colon cancer. ^c^TNM denotes tumor-node-metastasis. ^d^Detailed information pertaining to the receipt of postoperative adjuvant chemotherapy was available for 247 patients in the current study. Chemotherapy was primarily fluorouracil based with or without leucovorin, levamisole, or cisplatin. ^e^Detailed information pertaining to the receipt of intraoperative chemotherapy was available for 193 patients in the current study. The intraoperative chemotherapy regimens consisting of hydroxycamptothecin, calcium folinate (TNF), 5-fluorouracil (5-FU), and calcium folinate (CF) were administered during the operations. Hydroxycamptothecin was used as peritoneal irrigation during the operations, whereas TNF, 5-FU, and CF were given as intravenous injections. ^f^Standard of the 7th TNM stage of International Union Against Cancer (UICC) was used in our study

**Table 2:** Univariate and Multivariate Cox Proportional-hazard Regression Analysis of Receipt of IOC or POC and Cancer Survival in CRC Patients with Stage II or III^a^.

Characteristic	Univariate Analysis			Multivariate Analysis	
	HR (95% CI)	P Value		HR (95% CI)	P Value
**IOC**		<0.001			<0.001
Did not receive	1.0 (Reference)			1.0 (Reference)	
Received	0.33 (0.23-0.48)			0.30 (0.19-0.48)	
**POC**		0.006			<0.001
Did not receive	1.0 (Reference)			1.0 (Reference)	
Received	0.62 (0.45-0.87)			0.44 (0.30-0.64)	
**TNM stage**		<0.001			<0.001
I-II	1.0 (Reference)			1.0 (Reference)	
III-IV	2.52 (1.89-3.36)			2.35 (1.59-3.48)	
**Lymph node metastasis**		<0.001			0.412
No	1.0 (Reference)			1.0 (Reference)	
Yes	1.70 (1.28-2.25)			1.17 (0.80-1.72)	
**Age**		0.995			0.998
< 50	1.0 (Reference)			1.0 (Reference)	
≥ 50	0.99 (0.56-1.79)			1.00 (0.50-2.01)	
**Gender**		0.368			0.769
Female	1.0 (Reference)			1.0 (Reference)	
Male	0.88 (0.67-1.16)			1.05 (0.74-1.50)	
**Tumor location**		0.694			0.991
Colon	1.0 (Reference)			1.0 (Reference)	
Rectum	0.94 (0.70-1.27)			1.00 (0.70-1.44)	
**Tumor size**		0.350			0.894
< 5 cm	1.0 (Reference)			1.0 (Reference)	
≥ 5 cm	1.15 (0.86-1.52)			0.98 (0.69-1.38)	
**Differentiation**		0.024			0.018
Good	1.0 (Reference)			1.0 (Reference)	
Moderate or Poor	1.92 (1.09-3.36)			2.16 (1.14-4.06)	

Abbreviations: CI, confidence interval; HR, hazard ratio. ^a^Patients with TNM stage II or stage III cancer with typical carcinoma history were included in this analysis. ^b^P < 0.05 was considered to indicate statistical significance.

## Abbreviations

IOC: intraoperative chemotherapy
CRC: colorectal cancer
TNF: tumor necrosis factor
5-FU: 5-fluorouracil
CF: and calcium folinate
POC: postoperative adjuvant chemotherapy
